# Unraveling mitochondrial piRNAs in mouse embryonic gonadal cells

**DOI:** 10.1038/s41598-022-14414-4

**Published:** 2022-06-24

**Authors:** Odei Barreñada, Eduardo Larriba, Daniel Fernández-Pérez, Miguel Ángel Brieño-Enríquez, Jesús del Mazo Martínez

**Affiliations:** 1 Department of Cellular and Molecular Biology, Centro de Investigaciones Biológicas Margarita Salas C.I.B. (CSIC), Ramiro de Maeztu 9, 28040 Madrid, Spain; 2 Institute of Bioengineering, University “Miguel Hernández”, Elche, Spain; 3 Department of Experimental Oncology, IEO European Institute of Oncology–IRCCS, Milan, Italy; 4 Department of Health Sciences, University of Milan, Milan, Italy; 5 Magee-Womens Research Institute, Department of Obstetrics, Gynecology and Reproductive Sciences. School of Medicine, University of Pittsburgh, Pittsburgh, USA

**Keywords:** Cell biology, Developmental biology

## Abstract

Although mitochondria are widely studied organelles, the recent interest in the role of mitochondrial small noncoding RNAs (sncRNAs), miRNAs, and more recently, piRNAs, is providing new functional perspectives in germ cell development and differentiation. piRNAs (PIWI-interacting RNAs) are single-stranded sncRNAs of mostly about 20–35 nucleotides, generated from the processing of pre-piRNAs. We leverage next-generation sequencing data obtained from mouse primordial germ cells and somatic cells purified from early-differentiating embryonic ovaries and testis from 11.5 to 13.5 days postcoitum. Using bioinformatic tools, we elucidate (i) the origins of piRNAs as transcribed from mitochondrial DNA fragments inserted in the nucleus or from the mitochondrial genome; (ii) their levels of expression; and (iii) their potential roles, as well as their association with genomic regions encoding other sncRNAs (such as tRNAs and rRNAs) and the mitochondrial regulatory region (D-loop). Finally, our results suggest how nucleo-mitochondrial communication, both anterograde and retrograde signaling, may be mediated by mitochondria-associated piRNAs.

## Introduction

Mitochondria are the cellular “powerhouse” for ATP production. Theses organelles are crucial in the control of the energetic metabolic processes of the cell by different pathways^1^. In mammals, the vast majority of the hundreds of proteins involved in the structure and function of mitochondria are encoded by the nuclear genome. However, mitochondrial DNA (mtDNA) retains several key genes, including 13 key protein subunits involved in the electron transport chain complex, 2 rRNA subunits, 22 tRNAs, and the D-loop, a noncoding regulatory region involved in the control of replication and transcription of the mtDNA^2,3^.

Primordial germ cells (PGCs) are the precursors of the male and female gametes, which in mammals, after migration from the extraembryonic mesoderm to the gonadal ridges, initiate sex differentiation in proespermatogonia or oogonia /oocytes around day 12 postcoitum (dpc) in mice^4,5^. In humans, it has been observed that female PGC mitochondria increase in number and change morphology with their settlement in the genital ridge^6,7^.

Small non-coding RNAs (sncRNAs) play a key role as fine regulators of gene expression, including that affecting mitochondrial metabolism and function. MicroRNAs (miRNAs) and PIWI-interacting RNAs (piRNAs) are some of the most abundant types of sncRNAs. Mito-miRNAs have been the most studied and evaluated for their possible roles in development and pathogenesis^8–10^. piRNAs are single-stranded sncRNAs of mostly about 20–35 nucleotides (nt) that, in metazoans, actively participate in gene-silencing mechanisms through specific RNA interference pathways, with special focus on germline transposable elements (TEs)^11,12^. However, nowadays the presence of piRNAs in multiple somatic cells and species and functional roles, in addition to those related to TE interference, is well established^13–18^, including modulation of gene expression at the transcriptional or post-transcriptional level^19^ by interactions with different RNAs, such as mRNAs, transcribed pseudogenes, or long noncoding RNAs having, in part, similar mechanisms to those of miRNAs. Some approaches based on computational algorithms to identify sequences corresponding to piRNAs and potential specific functions, such as deadenylation of mRNAs, have been recently reported^20,21^. Dysfunctions of gene regulation piRNA-mediated interactions can lead to pathological consequences, including cancer ^22–24^. Emergent reports on non-transposon functions suggest new potential roles for piRNAs^25^.

The biogenesis processes of piRNAs have been established in various animal species, with elements common to most of them, in both germ and somatic cells^26–29^. The association of piRNAs with mitochondria has been well documented^30–32^. Moreover, the presence of piRNAs within the mitochondria has been detected^33^, although their biogenesis, variability, and potential functionality is still unclear. Furthermore, linkage between mitochondria and the piRNA biogenesis pathway has been clearly established on the basis of localization of the mitochondrial membrane-associated Zuc/MitoPLD protein, capable of hydrolyzing a mitochondria-specific lipid, cardiolipin; in turn, Zuc/MitoPLD is key to the biogenesis and functional activity of piRNAs^34–37^.

In previous studies, we have identified the widespread presence of mitochondria-associated piRNAs (mito-piRNAs) in mouse germ cells, zygotes, and somatic cells^38^. In the present work, we have analyzed diverse features of mitochondrial piRNAs in a differential developmental process, evaluating both germ and somatic cells in early mouse embryonic gonads, from 11.5 to 13.5 dpc. Annotation of piRNAs has been controversial since each database uses different parameters to annotate a sequence as piRNA. To avoid this confusion, we built and use a custom database piRNA-IPdb^39^ (https://ipdb2.shinyapps.io/ipdb2/), restricting piRNA selection criteria from the piRBase^40^ to those sequences that are immunoprecipitated, in mice, with any PIWI protein. This approach avoids the possibility of degradation products that were annotated as valid piRNAs.

Using these tools after next-generation sequencing (NGS) and bioinformatic approaches, we analyzed various features of the mitochondrial piRNAs. The extensive communication of mitochondria with the rest of the cell is a key regulatory mechanism in the overall cellular activity, including the regulation of gene expression as a consequence of mitochondria-nuclear communication. The mapping of sequences identified as mito-piRNAs due to their binding to PIWI family proteins, identified piRNAs that match exclusively in the mitochondrial DNA (MT) or in the nuclear regions related to the mitochondrial genome (NUMTs). The analysis of potential piRNA precursors enabled us to predict prospective nucleo-mitochondrial communication in gonadal cells during these early stages of development. Likewise, the sequence associations between identified mito-piRNAs with different genomic elements, such as mitochondrial D-loop, miRNAs, or other sncRNAs and tRNA fragments^41–43^, were assessed to illustrate the potential biogenesis pathways and interactions between piRNAs and diverse sncRNAs and their complex regulation associated with mitochondria. That is, we sought to discover the possible origin and functional roles of some mito-piRNAs.

## Results

### Global characterization of sncRNA sequences

In order to have a global view of the expression of both piRNAs and miRNAs, as well as their mapping on the mitochondrial genome (MT) or on both mitochondrial and nuclear genomes (NUMT), we have classified all reads following the stated criteria of mapping (see “Methods” section) and quantifying the number of sequences identified and the number of reads in each of the samples studied.

Reads of small RNAs from the different samples analyzed: primordial germ cells (PGCs) or somatic cells (SCs) for each developmental day and sex are shown in Table 1. This data, shows the relatively low proportion of all small RNAs matched with mitochondrial sequences (0.7% of reads from all sequences analyzed). But this low percentage equates to an overwhelming enrichment in the production of piRNAs per Kb of this relatively tiny mtDNA (Supplementary Fig. 1).

**Table 1:** Summary of unique the sequences (Seq) and reads (Reads) of sncRNAs mapping to genetic mitochondrial features, matching with miRNA and piRNA databases, differentiating those that map exclusively to the nuclear genome (NU), exclusively to the mitochondrial genome (MT), or to both genomes (NUMT). Note that (*) indicates other sncRNAs that, mapping to mitochondrial sequences, do not only correspond to miRNAs or piRNAs.

Samples	Total reads (> 10 CPM)		Mitochondrial Small-RNAs		miRNAs						piRNAs					
					NU		Mitochondrial				NU		Mitochondrial			
							NUMTs		MT				NUMTs		MT	
	Seq	Reads	Seq	Reads	Seq	Reads	Seq	Reads	Seq	Reads	Seq	Reads	Seq	Reads	Seq	Reads
PGC11F	8500	6,888,195	68	35,377	1514	1,838,954	1	730	0	0	5423	4,182,089	43	27,777	12	4479
PGC11M	8727	4,862,017	129	45,192	876	591,746	2	316	0	0	5356	2,782,782	76	30,371	20	7917
PGC12F	9297	5,926,773	52	18,489	454	262,577	0	0	0	0	5614	3,174,696	29	12,202	12	3859
PGC12M	9270	5,178,085	77	29,524	506	294,223	1	147	0	0	5538	2,760,811	45	22,131	14	4054
PGC13F	8901	5,058,940	100	42,942	892	627,937	0	0	0	0	5665	2,929,960	51	27,600	20	8828
PGC13M	8077	5,316,411	135	46,442	680	518,919	1	191	0	0	4769	2,936,741	74	27,668	23	10,728
SC11F	7124	5,700,359	79	32,770	2374	2,867,435	1	569	0	0	4830	4,039,697	48	24,720	13	3911
SC11M	8137	4,405,948	99	29,865	1246	892,796	1	267	0	0	5251	2,719,611	54	18,142	18	7246
SC12F	8033	5,928,611	56	21,317	2064	2,554,390	1	435	0	0	5101	3,717,637	35	16,990	8	1760
SC12M	8051	5,619,407	81	32,213	1922	2,233,411	1	499	0	0	5200	3,529,894	42	22,920	17	5229
SC13F	8093	6,712,405	138	64,297	2055	2,907,810	1	766	0	0	5246	4,456,638	79	46,651	23	8624
SC13M	7344	4,535,372	144	68,467	2117	1,722,383	1	438	0	0	4561	2,838,307	83	49,195	20	10,350
Average	8296	5,511,044	97	38,908	1392	1,442,715	1	363	0	0	5213	3,339,072	55	27,197	17	6415
Prop. of total reads:		100%		0.71%												
Proportion related to mito-small-RNAs				100%	*			0.9%		0.0%				69.9%		16.5%

To dissect the mitochondrial sncRNAs, we separated and analyzed miRNAs and piRNAs. We observed that only 0.9% of the reads corresponded to miRNAs, none of which are exclusively associated with the mitochondrial genome. Moreover, a very small number of sequences have been identified within the group of miRNAs mapping as NUMTs (Table 1). However, from the total mito-sncRNAs 86.4% of reads corresponded to piRNAs. It is noteworthy that 16.5% of the piRNA reads corresponded to sequences that matched exclusively to the mitochondrial genome. The remaining sequences were not identified as corresponding to either of these two groups: miRNAs or piRNAs (Table 1).

While, considering all identified sequences, the proportion of reads corresponding with miRNAs are higher in SCs than in PGCs, and the opposite is true for piRNAs. Taking into account only those associated with mitochondria, no significant differences were detected among the 12 samples analyzed, neither by cell type nor by developmental day nor by sex. This suggests that the mito-sncRNA specific expression pattern is common to all mouse embryonic gonadal cells in these early stages. Interestingly, in the sequence classification analysis, we have detected a group of sncRNAs that can be dually classified as both piRNAs and miRNAs; these will be considered in a later section.

**Figure 1:**
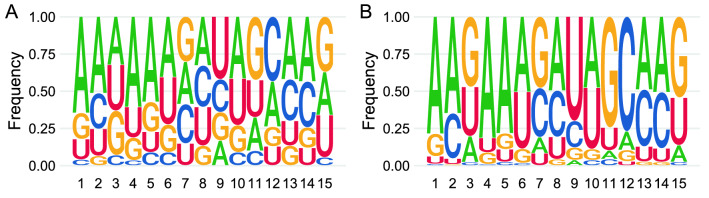
Sequence logo showing different bias signatures that were detected in mitochondrial piRNAs in our samples. (**A**) Counting all unique sequences. (**B**) Counting all reads.

Considering the canonical characteristics of piRNAs, we also analyzed the sequences of the mito-piRNAs. In this context, a classic established hallmark for piRNAs is the bias of the presence of uridine at the 5' position of piRNAs (1U), clearly detected in the overall analysis of the piRNA database used in the present study as reference for piRNA consideration (piRNA-IPdb)^39^. However, neither the sequences nor reads of the specific mito-piRNAs that we have identified in gonadal cells showed such a hallmark, so that, rather than the classical uridine at the 5′ 1 position (1U), adenine (1A) was found by sequences or by reads (Fig. 1).

### Localization of piRNAs mapping sequences in the genome

**Figure 2:**
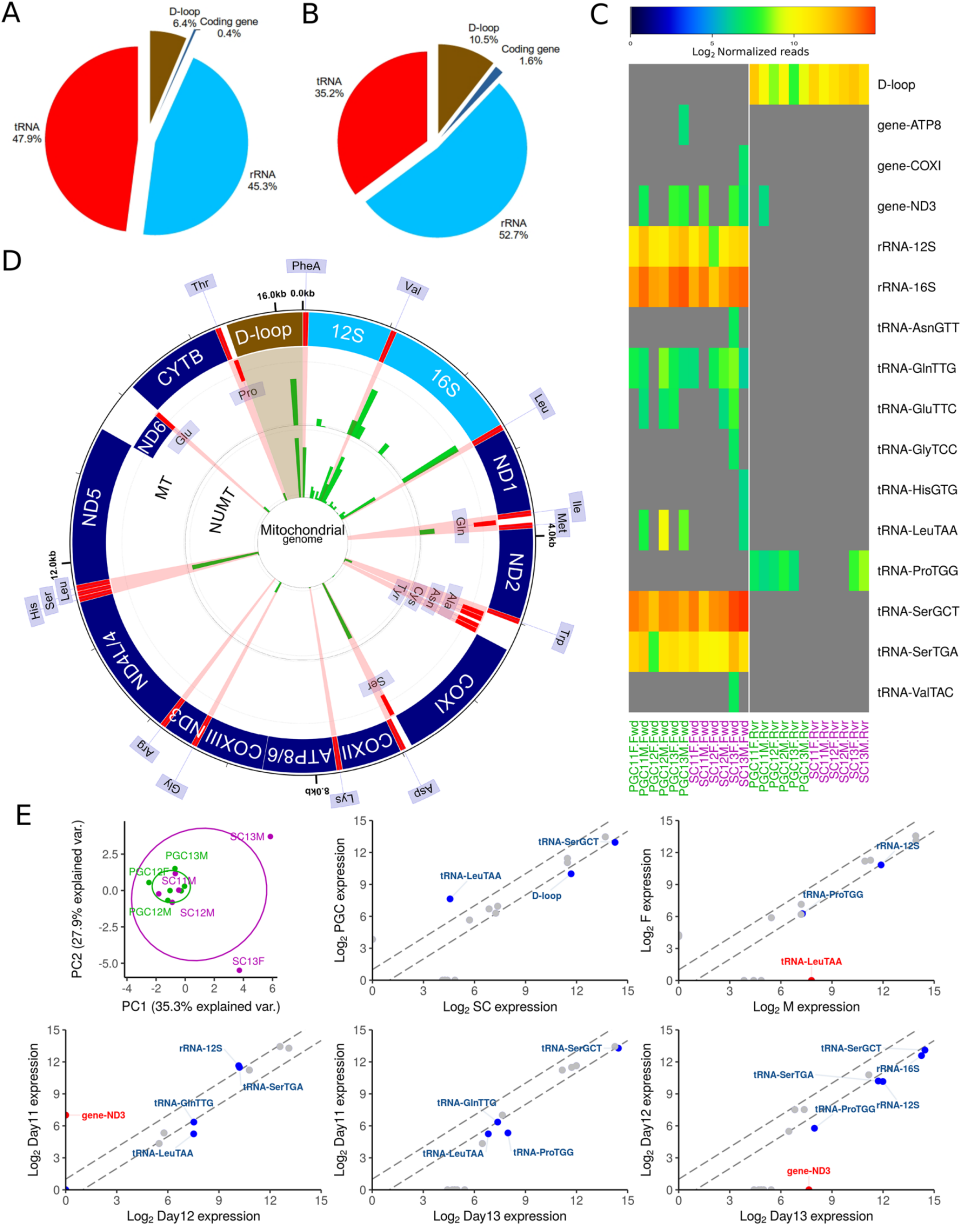
(**A**) Global feature distribution sequences of piRNAs matching regions related to the mitochondrial genome. (**B**) Global feature distribution of piRNA reads matching regions related to the mitochondrial genome. (**C**) Heatmap representing supervised hierarchical clustering overexpression of mito-piRNAs in forward (Fwd) and reverse (Rvr) orientation with respect to the mitochondrial genome in the samples analyzed. (**D**) Circular map of the level of expression of piRNAs mapping in the mitochondrial genome; MT and NUMT classified reads are plotted separately; tRNA and D-loop region are highlighted. (**E)** PCA and comparative analysis between cell types: PGCs vs SCs; sex: F *vs* M; and among the three developmental days assessed (11.5, 12.5, and 13.5 dpc). PGC: primordial germ cells. SC: somatic cells. F: females. M: males.

For mapping the detected mito-piRNA sequences in the genome, we have analyzed those that have been classified as MT, matching only to the mitochondrial genome, and those that also match mitochondrial sequences in the nuclear genome (NUMT). The majority of mito-piRNAs correspond to those regions encoding for tRNAs and rRNAs, both in number of sequences (Fig. 2A) and in the level of expression by number of reads (Fig. 2B). As shown in Fig. 2C, there are other regions of the mitochondrial genome where most of the piRNA reads correspond to sequences that match regions integrated into the D-loop region. The two rRNAs and some of the mitochondrial tRNAs are encoded in the mitochondrial genome, basically tRNA-Val, tRNA-Met, tRNA-Asp and tRNA-Ser. The protein-coding regions did not show significant reads matching piRNA sequences. Some regions of the mitochondrial genome only map piRNAs that uniquely match the mitochondrial genome (MT), such as that corresponding to tRNA-Met. It should be noted that there are broad regions where multiple piRNAs appear to be mapped, such as D-loop or 12S and 16S rRNAs. However, in these regions, specific MT piRNAs may coincide with sequences considered as NUMTs that, having the same 5' end, vary in length—what we refer as "families" of piRNAs, which are detailed below.

To evaluate the potential role of the detected mito-piRNAs associated with the mitochondrial genome, we have carried out analysis of the sequences separated by their mapping to the various features, in forward or reverse sense with respect to the coding sequence in the mitochondrial genome. The analysis performed in the different samples is shown in a supervised heatmap, which clearly indicates that, in the D-loop region, all the piRNAs detected correspond to the reverse direction as well as, in some samples (mainly PGCs), to tRNA-ProTGG (Fig. 2D). This could suggest a potential regulatory action, by generating an R-loop^44^ at the region of the D-loop and by interference with the tRNA-Pro transcript. In contrast, the most highly expressed piRNA sequences corresponding to rRNA sequences, together with tRNA-SerCGT and tRNA-SerTGA, are always in the forward direction, suggesting that tRNA-derived fragments (tRFs) should be the sources of such piRNAs. The expression of tRFs is part of the regulatory mechanisms, and consequently, the generation of piRNAs from tRFs by binding to PIWI proteins could be part of such regulatory pathways. Interestingly, no significant differences by cell type, sex, or time of development were detected. This has been corroborated by PCA analysis and by differential expression among the group of samples, in which only punctual mito-piRNAs with differential expression were significantly detected, as were those matching at the ND3 gene and tRNA-LeuTAA (Fig. 2E). No other groups show any clear differentiation (Supp. Figure 2).

### From mito-piRNA families to mito-nuclear communication

From the analysis of the total sequences obtained by NGS before being screened by our piRNA-IPdb database, we were able to detect multiple sequences corresponding to sncRNAs, longer than usual for piRNAs, which however, presented sequence-5’ homology with other piRNAs that were identified ,"*bona fide,*" as piRNAs in the piRNA-IPdb database. This suggested that the long sequences might correspond to precursors of piRNAs (pre-piRNAs), transcribed from the mitochondrial or nuclear genome, not yet processed by trimming from the 3' end^45–47^, and generate shortened mature piRNAs to be bound to PIWI proteins, already detectable in the piRNA-IPdb database. The analysis of all intermediate molecules from a potential primitive piRNA or pre-piRNA^48–50^, which we have called "piRNA families," could facilitate the characterization of the genomic origin (mitochondrial or nuclear) and the potential target of the piRNAs. The rationale of this analysis is based on the fact that if, for example, the longer pre-piRNAs, not detected as being bound to PIWI, match, in full sequence, with nuclear (NU) but not mitochondrial (MT) genomic sequences, the pre-piRNAs must have been transcribed unequivocally from the nuclear genome (NU), and the processed piRNAs (derived from each of these pre-piRNAs), matching both nuclear and mitochondrial (NUMT) genomes, could have targets at the mitochondrial or nuclear levels or both (as well as the opposite, if the sequence of such pre-piRNAs only matched that of the mitochondrial genome). That is, since piRNAs may act by binding to both RNA and DNA^47,51,52^, if the origin is exclusively mitochondrial (pre-piRNA matching only on mitochondrial DNA), it is tempting to hypothesize that the processed mito-piRNAs (shorter and binding PIWI) could act as a regulatory mechanism, in a retrograde way, at the level of the nuclear genome, where the sequences of such processed piRNAs match entirely with the nuclear DNA. Similarly, if the pre-piRNAs map to the nuclear genome, the processed piRNA can now match the mitochondrial DNA in an anterograde way.

**Figure 3:**
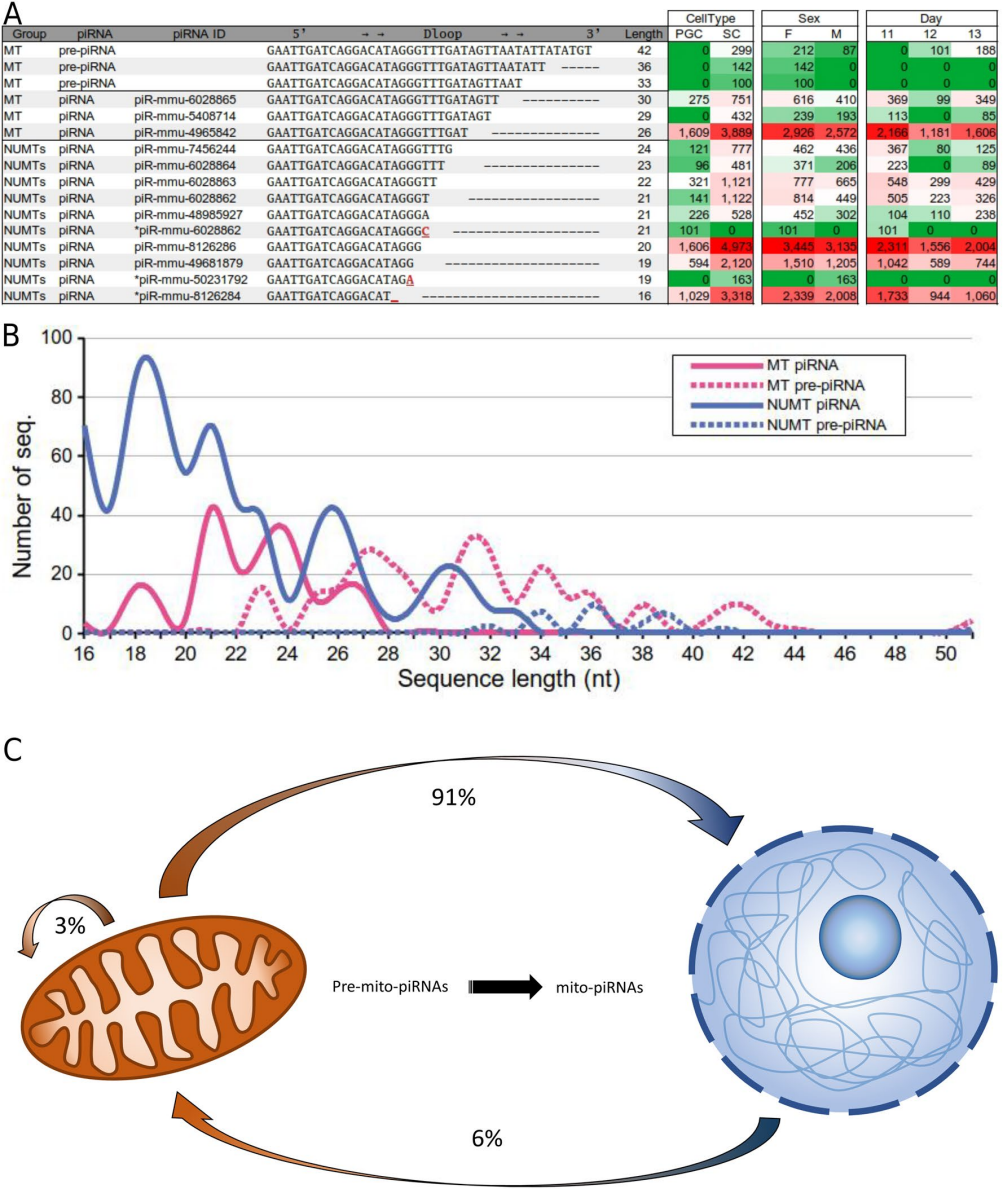
(**A**) Example of a family of mito-piRNAs matching pre-piRNAs exclusively in one region of the mitochondrial D-loop (mtDNA 16,121 to 16,189), where they are mapping multiple mito-piRNAs. The annotation column was added to show the sequence’s matching piRNA ID, and when multiple piRNAs are matched (with the allowed 1 mismatch), an “*” is added before the ID. The expression values are indicated in the attached table, sorted by cell type, sex, and day of development (marked from low, in green, to high, in red). (**B)** Distribution of mito-piRNAs and the corresponding pre-piRNAs in relation to sequence length mapping classified as MT or NUMT. (**C)** Illustration of expected proportion of mito-nuclear communication mediated by mito-piRNAs in retrograde, anterograde, or self-regulation pathways. The proportions were calculated as the percentages of total detected mitochondrial piRNA families (considering the detected genomic origin of each pre-piRNA and the potential target and were based on sequence complementarity of processed piRNA).

Figure 3A illustrates the basis for interpreting this nucleo-mitochondrial communication pathway in a defined region of the mitochondrial genome (D-loop) where abundant piRNAs were identified. The group of sequences considered pre-piRNAs (not detected as associated with any PIWI protein) are always exclusively mitochondrial (MT), while the short sequences have multiple mapping (mitochondrial and nuclear, NUMTs). All these sequences, which are processed by 3' shortening and binding to PIWI form what we have called a family of piRNAs. Evaluating the expression values of the corresponding identified sequences, no significant changes were detected among the samples, which strongly suggests that this pattern could be constitutive and not dependent on the cell type, sex, or developmental stage.

Globally, comparing the distribution of sequence lengths in both MTs and NUMTs in relation to their expression levels, we observe a clear difference in the proportion of exclusively mitochondrial (MT) pre-piRNAs, while NUMT piRNAs are mostly of the mature piRNA type (Fig. 3B).

As a consequence of this analysis, evaluating the pooled data of all the cell samples, it is possible to suggest that, at least in gonadal mouse cells (PGCs and SCs), the piRNA-mediated communication is mostly of the retrograde type; that is, from pre-piRNAs generated in the mitochondria, with potential regulatory action on the nuclear genome level. The minority anterograde type of regulation, from the nucleus to the mitochondria, is illustrated in Fig. 3C. In most of the samples, there are at least three families of conserved piRNAs, corresponding to the regions: tRNA-SerGCT; 16S rRNA (at both ends); and D-loop.

### mito-piRNA and miRNA duality

**Figure 4:**
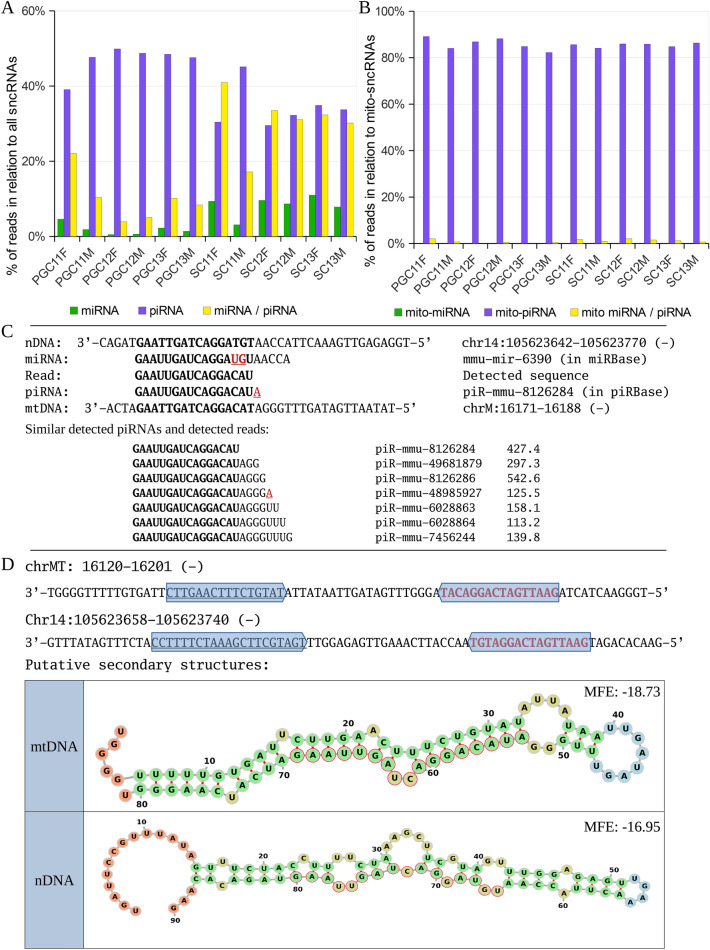
(**A**) Distribution of miRNAs, piRNAs, and dual miRNA/piRNAs in the samples analyzed from both germ (PGCs) and somatic cells (SCs) from the total of sequenced sncRNAs. (**B**) The same distribution corresponding only to the mito-sncRNAs. (**C**) Mapping of mmu-mir-6390/piR-mmu-8126284 and other related mito-piRNAs in both nuclear and mitochondrial genomes. The read counts are expressed as means between all samples. (**D**) Potential secondary RNA structure of the region containing mmu-mir-6390/piR-mmu-8126284 sequences. The Minimum Free Energy (MFE) is expressed in kcal/mol and calculated by RNAfold software with custom parameters.

By comparatively analyzing all sequences, we have detected a group of mito-piRNAs that show a dual matching with miRNAs in the corresponding database. In order to establish this possible duality of sequences identifiable as piRNAs and, at the same time, as miRNAs, we first assessed this situation in the set of all sncRNAs in the various samples of our study (Fig. 4A). The ratio of piRNAs to miRNAs is clearly higher, as expected, given that piRNAs are the most abundant class of sncRNAs^53^. Interestingly, in addition to the proportional increase of miRNAs in SCs with respect to PGCs, those sequences that can be categorized as both miRNAs and piRNAs are also much more abundant in SCs (Fig. 4A). However, no miRNAs were identified as mitochondria specific (Table 1 and Fig. 4B), and those considered to have dual mito-miRNA/mito-piRNA characteristics were very scarce (Fig. 4B). Such dual potential suggests that some of miRNAs might also bind PIWI proteins.

To verify the putative biogenesis of this dual situation, we evaluated the genomic sequences around the each specific mito-miRNA/mito-piRNA to detect the structure necessary to generate a classical pri-miRNA and pre-miRNA with a putative double-strand region as a biogenic hallmark. Although having very limited representation as mito-miRNAs/mito-piRNAs, the most frequent sequence detected corresponds to mmu-mir-6390/piR-mmu-8126284 (as well as other piRNAs with a wide level of matching in the mitochondrial genome) (Fig. 4C). By analyzing its potential biogenesis on the basis of the sequences that match the nuclear and mitochondrial genomes, we have detected that both situations are possible. That is, this miRNA and this piRNA can be generated from either genome, mitochondrial or nuclear. However, the identification of similar sequences of other piRNAs matching better with the mitochondrial genome (Fig. 4C) would suggest that the piRNAs would be of mitochondrial origin and the miRNA from one or the other genome, as could be determined on the basis of the stability of the secondary structure after transcription from either the nuclear or the mitochondrial genome (Fig. 4D). Interestingly, mmu-mir-6390 is highly expressed in mouse embryonic gonads (MGI database: http://www.informatics.jax.org/).

## Discussion

piRNAs are not only the most abundant class of sncRNAs, but probably the most complex, both in their biogenesis and in their potential multiple functional roles^54,55^. It is now evident that the presence and activity of piRNAs is not unique to the germline^56,57^. Their expression patterns and activity in various animals are also being considered as key elements in these functions. We and others have previously characterized mito-piRNAs in diverse germ cells^12,38^; however, these mito-piRNAs are not exclusive to the germ cell line since their presence in somatic cells has also been described^33^. Mitochondria had been associated with piRNAs initially because of the presence of proteins in the mitochondrial membrane involved in piRNA biogenesis^47,49,50,58–60^. However, the dynamics of piRNA biogenesis and its association with the mitochondrial genome, as well as the action of piRNAs in mitochondria, have not been fully established. With NGS of small RNAs and bioinformatic analysis, we were able to show some aspects of both biogenesis, potential function, and piRNA-mediated mito-nuclear communication. For a comparative analysis, we have analyzed, as a model, the two cell types, somatic and germ cells, during early gonad development in a key period for male and female germ line differentiation.

The feature and bias of uridine as the first nucleotide of piRNAs (1U) was generally associated with activity against transposable elements (TEs) and amplification through the "ping-pong" mechanism. Nevertheless, our data for mito-piRNAs do not indicate such a bias. The mitochondrial genome lacks elements properly considered as transposons, and consequently, this feature would not be expected. Nevertheless, a bias toward adenine as the first nucleotide is evident in the mito-piRNAs. This characteristic of mito-piRNAs, which we have already detected previously in other cell types, including spermatogonia cells, spermatozoa, oocytes, and zygotes, suggests that the functionality and biogenesis of the mito-piRNAs identified in these cell types, and putatively in other cells, may have different relations than those classically established to interactions with TEs and the "ping-pong" pathway^61^. On the other hand, it has been demonstrated that the feature of 10A in secondary piRNAs, after amplification by the ping-pong pathway is not a direct consequence of the presence of uridine in position 1 of the primary piRNAs but is a consequence of the preference of the PIWI protein, which in the case of MILI, is adenine in the target molecule, which has a binding pocket in its structure that best accommodates adenine in position 1^62^. It has also been recently proposed that PIWI proteins discriminate against RNA molecules with first nucleotide C or G, with a preference for A/U-richness of piRNA precursors^63^. This suggests that the characteristics of different types of piRNAs might be associated with their functional mechanism of action. The peculiar feature of 1A bias in mito-piRNAs should be verified in other cell types.

The association of piRNAs with both tRNAs (or tRFs) and rRNAs in germ cells has been previously reported^30,64^. The generation of tRFs as a consequence of the processing of mature or premature tRNAs^65,66^ is currently considered a mechanism of regulation, not a simple product of degradation^43^. Given the profusion of genes and pseudogenes coding for tRNAs and rRNAs and their evolution, in some cases, to repetitive elements and TEs coding in the genome of vertebrates^67^, the existence of piRNAs associated with them is not surprising. The involvement of tRFs in cellular processes is now generating emerging interest both in differentiation and development and in pathogenesis^18,41^. The mitochondrial genome contains coding sequences for key functional tRNAs. The identification in all samples of mito-piRNAs matching tRNA sequences in the mitochondrial genome, especially tRNA-Ser (forward sense) (Fig. 2), suggests a differential function of the tRNA-associated mito-piRNAs that merits further investigation. Considering that we have identified piRNAs as those RNAs bound to PIWI proteins, we do not agree that some of such molecules, including those that match with tRNA sequences, should be considered as artifactual contaminants of some databases^68^. In fact, in humans, piRNAs derived from tRNAs (bound to the human PIWI orthologue *HIWI2*) are detected both in somatic cells and in testis^42^. Likewise, the abundance of mito-piRNAs associated with rRNAs is a clearly remarkable feature. We could consider that both types of molecules, tRNAs and rRNAs, are key in the regulation of mitochondrial transcriptional activity. The participation of PIWI proteins and piRNAs in translation control have been remarked, in other studies, in both germline^38, 69–71^ and somatic cells^42^ and, more recently, in our previous analyses of non-mitochondrial piRNAs in these gonadal cell types^64^. Unfortunately, some recent reports, looking for piRNAs as biomarkers for specific pathologies, such as invasive breast cancer, remove mitochondrial and tRNA-associated sequences from the analysis^10^. As we show in the present study, piRNAs associated with such other sncRNAs (tRNAs and rRNAs) can expand our understanding of the role of piRNAs in such pathologies.

The mito-piRNAs can target the chromatin from which they are derived, perhaps, involved in such functions as opening the chromatin structure, as has been suggested for other types of sncRNAs detected in *C. elegans*, called 21U-RNAs, which in fact, have clear piRNA characteristics^72^. It is interesting to note that the mitochondrial region within the D-loop, where we have detected high concentrations of matching mito-piRNAs, corresponds to the initiation region of mitochondrial DNA transcription of the heavy and light chains (H and L-strains), as well as the origin of heavy chain replication (O_H_-strand)^3^. This strongly suggests that such mito-piRNAs generated through pre-piRNAs, as we have identified as a family of piRNAs (Fig. 3), would constitute a specific mechanism of transcriptional and replication regulation of the mitochondrial genome^30^, probably through the generation of an R-loop, with the inclusion of such piRNAs in the structure of DNA-RNA as was reported in early studies^73^. The participation of RNAs in regulation by binding to DNA by invading its double helix, not only in replicative intermediates, where the RNA primes or templates of DNA synthesis, have already been pointed out some time ago^74^. In fact, recent experiments demonstrate that some proteins, such SOX2 (participating in pluripotency reprogramming activity), associate with RNA and DNA simultaneously forming RNA/Sox2/DNA complexes^75^. Perhaps, the participation of piRNAs in the mitochondrial D-loop may be a good example of it.

Different noncoding RNAs have been reported to mediate bidirectional crosstalk and gene regulation between mitochondria and nucleus and vice versa^76–79^. This also includes some tRNA fragments^78^, which are very well represented in the piRNA sequences reported here. Crosstalk between mitochondria and nucleus can be mediated by specific proteins and ncRNAs^77,80–82^. Based on the classification and analysis of mito-piRNA families and their matching as MT or NUMT (see Methods), we can infer that the communication between mitochondria and the nucleus, piRNA-mediated, is mainly of the retrograde type (Fig. 3), which could be involved in special pathological processes^83^. The rationale for the interpretation of this process is based on the fact that the detection of long sncRNA molecules detected via NGS (considering the evaluation of sequences up to 75 nt in length), which although they did not align with sequences from the piRNA-IPdb, did match with the mitochondrial or nuclear genome. The existence of sequences shortened at the 3' end and considered as piRNAs in the piRNA-IPdb strongly suggested that those longer sequences would be precursors of the piRNAs (pre-piRNAs) matching in the mitochondrial and/or nuclear genome.

It is accepted that NUMTs are a consequence of mtDNA fragments integrated into the nuclear genome^84^. Analysis of the characteristics of NUMT sequences does not indicate the possibility that RNA elements such piRNAs could generate the integration of the sequences into nuclear DNA as NUMTs by cDNA intermediates^85^. Therefore, MT mito-piRNAs would not be the origin of NUMTs. Interestingly, it is controversial whether the D-loop region of the mitochondria, which is rich in mapped mito-piRNAs, is hardly represented in the nucleus as NUMTs^86,87^ or whether it is highly represented^88^. In any case, mitochondrial DNA fragments, integrated into nuclear DNA as NUMTs, could act on mitochondrial DNA or on their corresponding transcripts, modulating them in an anterograde mito-piRNA-mediated manner. These mechanisms could have important roles in pathogenesis (e.g., cancer)^85^. The fact of detecting pre-piRNAs not bound to Piwi but with 5' regions identical to piRNAs considered mature and bound to Piwi could be due to either: a) pre-piRNAs not being detected in the Piwi immunoprecipitation database (piRNA-IPdb) because, in most of the datasets, piRNAs longer than those considered of canonical length are not included; or b) such pre-piRNAs not having yet bound to Piwi proteins. In any case, the existence of such piRNA families can be considered a good marker to evaluate the origin and possible fate of mature piRNAs and, consequently, anterograde or retrograde signaling, mediated by mito-piRNAs, on mitochondria.

We have detected a clear example of such a family of mito-piRNAs in the mitochondrial D-loop region (Fig. 3). An interesting and recent report, supporting the existence of such mito-piRNA families and their importance in the retrograde regulation of the mitochondrial D-loop region, is the identification of an mtDNA-encoded ncRNA previously known as miR-805, which is differentially expressed in alveolar epithelial type II (AETII) cells and increased in the lungs of mice exposed to cigarette smoke. Such mito-ncR-805 with an RNA sequence of 70 bp, corresponds with a specific mitochondrial region MT (16,188–16,119)^31^. The mito-pre-piRNAs and all the family of piRNAs identified in our samples mapping in the D-loop region (Fig. 3A) share the same sequence from the 5' terminus of the mito-ncR-805 (Supplementary Fig. 3). This indicates that these biogenic pathways, at least in the D-loop, are not restricted to gonadal cells and could have a wide functional impact.

The potential interaction between mitochondria, piRNAs, and nucleus has some cytological and molecular manifestations. In germ cells, mitochondria are usually located in groups associated with granular structures, termed "nuage"^89^ or "intermitochondrial cement,"^90^ which are particularly enriched in piRNAs and proteins associated with the piRNA pathway^89^ and frequently found in close proximity to the nuclear pores^91^. Provocatively, these structures might suggest some communication with the nucleus, potentially piRNA-mediated. On the other hand, outer mitochondrial membrane and nuage agglutinate multiple elements of the primary piRNA biogenesis pathway, such as the proteins MITOPLD^91^**,** MVH^92^, and TDRKH^93^, associated with PNLDC1^55,56,94^ and MOV10L1. Specifically, MOV10L1 binds and unwinds piRNA precursors to generate piRNA precursor intermediates and mature piRNAs^95,96^.

The existence of sncRNAs with dual potential functions, such as miRNAs or piRNAs, although not substantial in number of sequences detected, is an observation to be considered functionally in the future. The mito-piRNAs might target the chromatin from which they are derived, perhaps, involved in such functions as opening the chromatin structure, as has been suggested for other types of non-canonical sncRNAs, such as 21U-RNAs, detected in *C. elegans*, which are not fully classified as miRNAs, but have clear piRNA characteristics^72^. An example of this potential dual scenario would be the sequence known as mmu-mir-6390/piR-mmu-8126284, whose duality can also be associated with its genomic origin, both in mouse chromosome 14 and in the mitochondrial genome. This miRNA/piRNA arises from hairpins characteristic of miRNA precursors (Fig. 4). However, the piRNA-IPdb identifies it as a piRNA and the mirBase as a miRNA. The existence of other mito-piRNAs mapping to the mitochondrial genome seems to favor piRNA functionality, but the other option cannot be ruled out. It is tempting to speculate that the potential duality of some sncRNAs could be related to the ratio or abundance of AGO proteins involved in the miRNA pathway *versus* PIWI involved in the piRNA pathway, in a competitive way as different AGO proteins compete for miRNAs^97^. However, the relatively high abundance of dual miRNA/piRNA in the analysis of all sncRNAs, not specific for mitochondrial sequences (Fig. 4A), could have an alternative explanation. This could be a consequence of erroneous ascription of piRNA sequences as miRNAs in the miRBase (i.e., miRNAs that have not been curated by functional assays) or miRNAs that have been erroneously integrated into the piRNA databases. Consequently, in all cases of possible miRNA/piRNA duality, analysis of the potential pre-miRNA structure should be carried out. Functional assays will, in the future, give a definite answer to this dynamic interaction between piRNAs and miRNAs.

In previous studies, we had reported the existence of differences in embryonic gonads depending on the cell type, both for miRNAs^98^ and for piRNAs^64^ However, specifically for mito-piRNAs, the comparative data between the different samples analyzed indicate that no significant differences dependent on sex, developmental stage or cell type (PGCs or SCs) are significatively detectable. Consequently, we can hypothesize that the characteristics reported here of mito-piRNAs may be independent of cell type or developmental stage, at least in mouse embryonic cells. It will be interesting to evaluate whether they are maintained in other cell types or could be molecular biomarkers of pathogenesis, especially in diseases related to mitochondrial activity.

## Methods

### Biological samples

Pregnant *Mus musculus* of the CD1 strain were used for us to dissect the gonads from embryos at 11.5, 12.5, and 13.5 dpc, following the protocols and animal care as described in Fernandez-Perez et al.^98^ All procedures relating to the care and handling of the mice were carried out in the CIB-CSIC bioterium under specific pathogen-free (SPF), temperature (22 °C ± 1 °C), and humidity-controlled (50%–55%) conditions. All animals were housed on 12-h light–dark cycles with ad libitum access to food and water.

Male and female gonads were processed independently. The gonadal sex at 11.5 and 12.5 dpc were identified by PCR in each embryo from somatic tissues, using primers to the genes *Sry* and *Jarid1d* (a single band for XX and a double band for XY were detected); at 13.5 dpc, gonadal sex was morphologically identified. PGCs and SCs were purified and separately collected by paramagnetic procedures as indicated in Fernandez-Perez et al.^98^ Briefly, the whole gonads were trypsinized and incubated with specific PGC antibodies (anti-CD15, Miltenyi Biotec) bound to paramagnetic microbeads. The cells were passed through a magnetic column and the purity checked by PGC-specific staining with As-MX/FAST-RED (Sigma-Aldrich) on both fractions. No samples under 95% purity were used. Groups of about 80 embryo gonads from about 10 pregnant mice were used in each cell-separation procedure.

### RNA isolation and small RNA sequencing

The embryonic gonads of the offspring of at least four pregnant mice of each situation were pooled to obtain enough RNA for sequencing. Total RNAs were purified by TRIzol Reagent (Invitrogen) following the manufacturer’s instructions. RNA concentrations and quality were measured on an ND-1000 spectrophotometer (NanoDrop) and in a 2100 Bioanalyzer (Agilent), respectively. In all RNA used, the RIN value of RNA integrity was over 8. To select small RNAs for sequencing, total RNAs were fractioned by electrophoresis in acrylamide gels to isolate fractions under 200 nt as a standard protocol, followed by commercial sequencing for the NGS process (BGI, China) using the current small RNA sequencing procedure. The 12 different samples were sequenced using at least 1 µg of total pooled RNA. After adapter ligations the RNA molecules followed by reverse transcription; MiSeq Sequencing System (Illumina) was used in single-end mode, with a read length of 75 bp and an average depth of 10 M reads (Supp. Figure 4).

### Bioinformatic analyses

The bioinformatic workflow is summarized in Supplementary Fig. 4. After analysis of the quality of the sequence data and filtering those sequences with low expression level to focus the results on the most representative sequences (those under 10 counts per million reads), two successive classification procedures were performed—by genome mapping and by sncRNA identification—to finally assess the characteristics of the detected mito-piRNA molecules.

### Sequencing quality assessment

The raw sequences obtained from short RNA sequencing were trimmed to eliminate the used adapter sequences (provided by the sequencing facility), discarding sequences under 28 Phred score, using Trim Galore custom script in version 0.4.1 with parameters: “-f fastq -e 0.1 -q 28 -O 1 -a AGATCGGAAGAGC” (www.bioinformatics.babraham.ac.uk/projects/trim_galore). The trimmed sequences’ quality was assessed by FastQC software (www.bioinformatics.babraham.ac.uk/projects/fastqc), ensuring the correct processing of the trimming. Clean sequences were collapsed using the FASTX-Toolkit suite (http://hannonlab.cshl.edu/fastx_toolkit/index.html). Additionally, to enrich the most representative sequences, all the sequences under 10 counts per million (CPM) were discarded, which is more restrictive, by one order of magnitude, than those considered in previous studies^99,100^. That is, 10 CPM were equivalent to 66–105 reads per sequence, depending on the total reads in each sample.

### Sequence annotation

The sequences were classified by both genome mapping and by sncRNA identification. As the first aim was to identify those sequences associated with any mitochondrial feature, we mapped all sequences that match exclusively in the mitochondrial genome (MT) or in the mitochondria but also in the nuclear genome (nuclear-mitochondrial sequences or NUMT). For this purpose, sequences were aligned using Bowtie v1.3 with two independent databases: the nuclear DNA (nDNA) GRCm39/mm39 database (www.ncbi.nlm.nih.gov/assembly/GCF_000001635.27) and the actual gold standard of mouse mitochondrial DNA sequence^101^ with GenBank ascension number AY172335 . In both cases, one mismatch between sequence and reference genome was allowed; the additional parameters passed to bowtie were: “-p 7 -v 1 –best –strata -y –chunkmbs 256 –sam”.

By Bowtie aligner, two types of sncRNAs, miRNAs and piRNAs, were identified. Using miRBase version 22.1^102^, allowing two mismatches for miRNAs, and a custom database, to consider only those mouse sequences identified in the piRBase^40^ detected after immunoprecipitation with PIWI proteins; the custom database, called piRNA-IPdb^39^, allowed 1 mismatch for piRNAs. The exact command passed to Bowtie was “-x < Bowtie Index of database > -f < Sample > -p 7 -v < mismatch > –sam-RG map_$mapping –best –strata -y –chunkmbs 256 –sam”. Items between “ <  > ” marks depend on database and/or sample. These parameters ensure the best possible match (–best –strata), avoiding lazy behaviours (*-y*) with extended memory cache (–chunkmbs).

We have considered as piRNA precursors (pre-piRNAs) those sequences detected from total sequences in our NGS approach which show a sequence identical to an identified piRNA at the 5' end but were, however, longer sequences (and not present in the piRNA-IPdb) and mapped entirely to the mitochondrial or nuclear DNA sequence.

### Sequence analysis

First, the mitochondrial mapping and associated features of all mito-piRNAs (NUMTs and MTs), taking as reference the GenBank: AY172335.1, were assessed. NUMTs are nuclear mitochondrial DNA segments integrated during evolution into the nuclear DNA^103,104^. These include tRNAs, rRNAs, OXPHOS machinery genes, and the regulatory D-loop region. In order to search potential dual functions, we have also identified sequences that can be classified as both miRNA (at miRBase) and piRNA (following the mentioned criteria).

### piRNA families

As the existence of groups of piRNAs having the same 5' end but exhibiting variable trimming at the 3' end can be clearly detected in the NGS carried out, we are terming "family" of piRNAs. Those having a common longer RNA precursor (pre-piRNAs) displayed different piRNAs matching at 5' with the pre-piRNA. We used CD-HIT software with a full match between shorter sequences and any 3’ extension (in the pre-piRNA) matching with the corresponding genome sequence. The specific CD-HIT command for piRNA family generation was “cd-hit-est -c 1 -M 0 -sc 1 -sf 1 -d 0 -gap 12 -gap-ext 6”. The concept of a piRNA family is different from the concept of a "cluster," typically ascribed to piRNAs, in which piRNAs with different sequences have widely (20–100 KB) defined loci^105^ transcribing very large precursor RNAs that are processed into primary piRNAs.

### Mito-piRNA mitochondrial-nucleus communication

To investigate the origin of genome-encoding precursors of piRNAs (pre-piRNAs), we analyzed, from total sequences obtained by NGS, those sequences that were longer than those considered as regular piRNAs and were not identified in the piRNA-IPdb database but contained 5' regions with complete homology to piRNAs included in piRNA-IPdb. All mature piRNAs with sequence homology to the specific pre-piRNA but bound to PIWI (referenced by the piRNA-IPdb) were included in the same piRNA "family." The sequences of the corresponding pre-piRNAs were compared with nuclear and mitochondrial DNA to identify in which of the two genomes their coding and transcriptional origin was located (NU, NUMT, or specifically MT). We evaluated the mapping of the identified piRNAs with one or other genome (MT or NUMT), to consequently consider the origin and the potentially fate of the piRNAs: mitochondria to nucleus or vice versa. In order to assess the mitochondrial family communication direction, we count families with at least three different sequences detected, with at least one of each piRNA class (pre-piRNA and mature piRNA) and with at least one of sequences classified as NUMT or MT.

### Ethics

This study was carried out following the Spanish Royal Legislative Decree RD53/2013 for the Care and Use of Laboratory Animals. The protocol was approved by the Committee on the Bioethics of Animal Experiments of the Centro de Investigaciones Biológicas (CSIC) and authorized by the Comunidad Autónoma de Madrid (CAM) (Ref PROEX: 054/15). All the procedures/methods in this research were carried out in accordance with ARRIVE guidelines.

## Supplementary Information

Supplementary Information 1.

Supplementary Information 2.

Supplementary Information 3.

Supplementary Information 4.

Supplementary Information 5.

## Data Availability

The RNA-seq raw data and count data discussed in this publication were deposited in NCBI’s Gene Expression Omnibus (GEO) and are accessible through GEO Series accession numbers GSE98713 and GSE179299 for raw and count data, respectively. Other information is available from the corresponding author on reasonable request.
