# Supplementary figures and images for: Unraveling mitochondrial piRNAs in mouse embryonic gonadal cells
Source: Sci Rep. 2022 Jun 24;12:10730. doi: 10.1038/s41598-022-14414-4 (PMC9232517; doi:10.1038/s41598-022-14414-4)

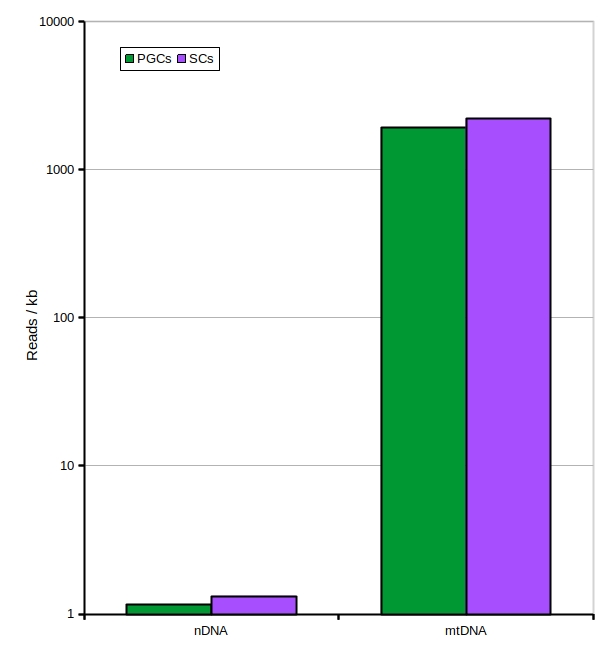

Supplement: Supplementary file 1 — Supplementary Information 1. [file 41598_2022_14414_MOESM1_ESM.jpg]

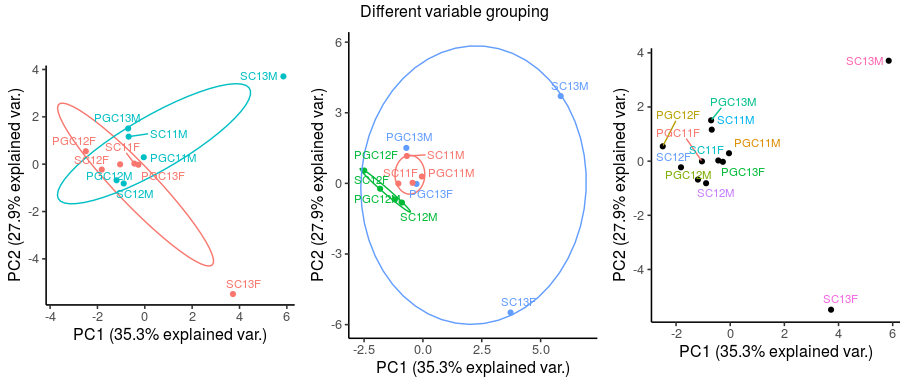

Supplement: Supplementary file 2 — Supplementary Information 2. [file 41598_2022_14414_MOESM2_ESM.tiff]

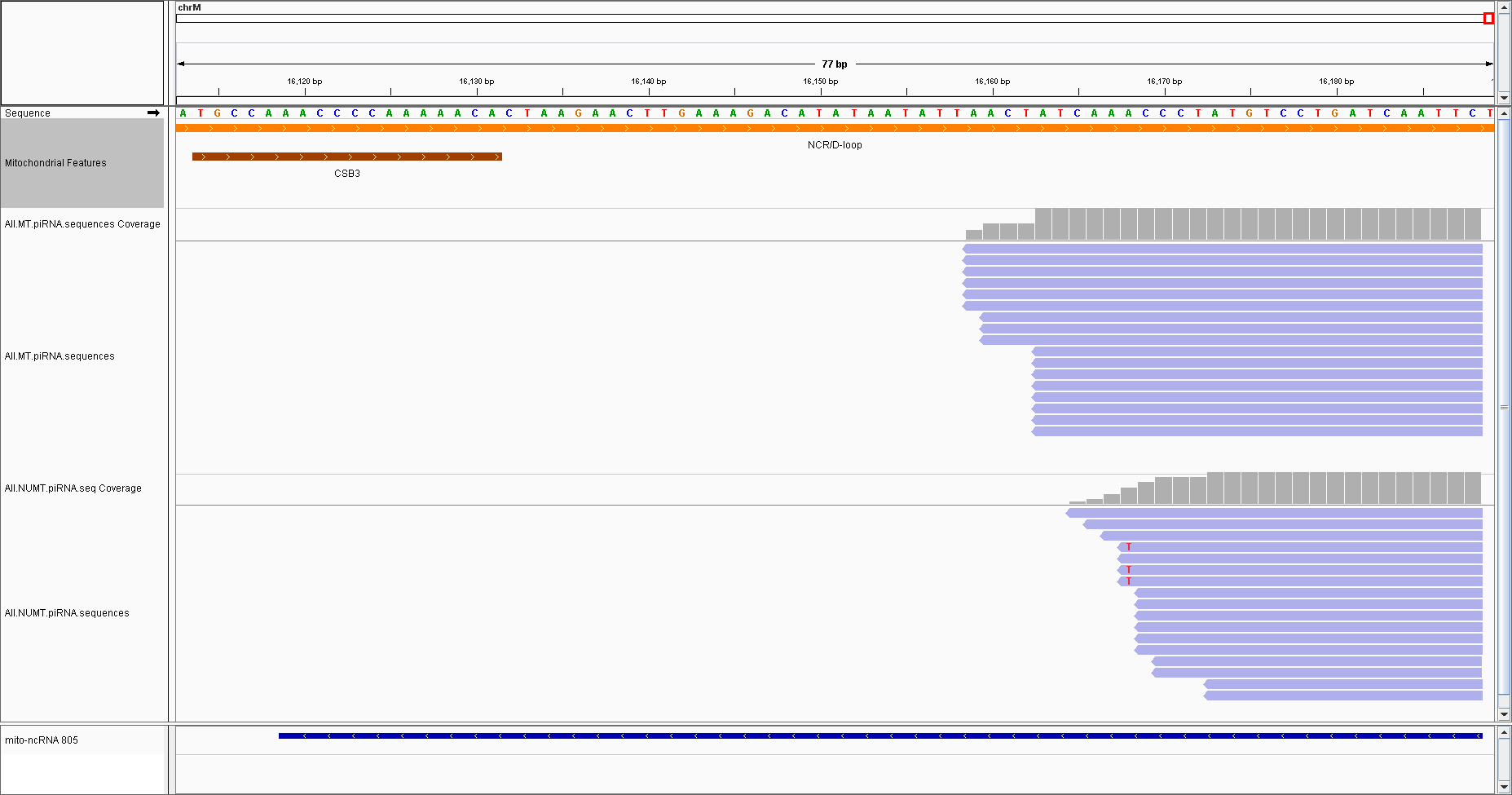

Supplement: Supplementary file 3 — Supplementary Information 3. [file 41598_2022_14414_MOESM3_ESM.jpeg]

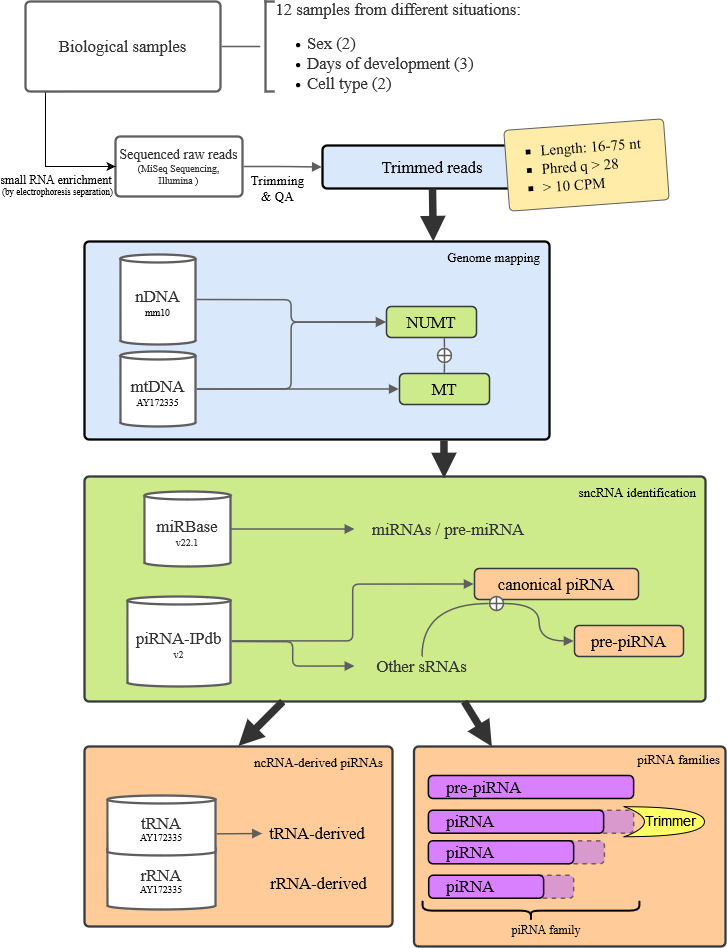

Supplement: Supplementary file 4 — Supplementary Information 4. [file 41598_2022_14414_MOESM4_ESM.jpg]
