# Supplementary material for: Unraveling mitochondrial piRNAs in mouse embryonic gonadal cells
Source: Sci Rep. 2022 Jun 24;12:10730. doi: 10.1038/s41598-022-14414-4 (PMC9232517; doi:10.1038/s41598-022-14414-4)
Supplement: Supplementary file 5 — Supplementary Information 5. [file 41598_2022_14414_MOESM5_ESM.docx]

**Supplementary Figure legends**

**Supplementary Figure 1**. Enrichment of piRNA production per kilobase at nuclear DNA and mitochondrial DNA.

**Supplementary Figure 2**. PCAs showing the lack of sample grouping depending on other variables such as sex (Males vs Females), development day (E11.5 vs E12.5 vs E13.5); and without groups.

**Supplementary Figure 3**. Comparative mapping at the mitochondrial genome in the D-loop region of mito-ncR-805 with sequences detected at our analysis (MT sequences as first track and NUMT sequences as second track). Exact coordinates are mtDNA 16114-16189.

**Supplementary Figure 4**. Workflow chart of the bioinformatic analyses.
